# Assessing the impacts of the Agenda Gap intervention for youth mental health promotion through policy engagement: a study protocol

**DOI:** 10.1186/s13033-020-00390-7

**Published:** 2020-07-31

**Authors:** Emily Jenkins, Rebecca Haines-Saah, Liza McGuinness, Saima Hirani, Noah Boakye-Yiadom, Tanya Halsall, Robert Rivers, Jonathan Morris

**Affiliations:** 1 School of Nursing, University of British Columbia, T201-2211 Wesbrook Mall, Vancouver, BC V6T 2B5 Canada; 2 Department of Community Health Sciences, Cumming School of Medicine, University of Calgary, 3280 Hospital Drive NW, Calgary, AB T2N 4N1 Canada; 3 The Royal’s Institute of Mental Health Research, 1145 Carling Ave, Ottawa, ON K1Z 7K4 Canada; 4 Senate of Canada, Chambers Building, C112-40 Elgin Street, Ottawa, ON K1P 1C7 Canada; 5 Canadian Mental Health Association, BC Division, 905-1130 West Pender Street, Vancouver, BC V6E 4A4 Canada

**Keywords:** Youth engagement, Mental health promotion, Policy, Community-based research, Realist evaluation

## Abstract

**Background:**

Mental health challenges are a leading health concern for youth globally, requiring a comprehensive approach incorporating promotion, prevention and treatment within a healthy public policy framework. However, the broad enactment of this vision has yet to be realized. Further, mental health *promotion* evidence specific to youth is still emerging and has not yet focused at a policy level. This is a critical gap, as policy is a key mental health promotion lever that can alter the social and structural conditions that contribute to shaping youth mental health outcomes for *all* youth, across the full spectrum of need. Responsive to this research and intervention priority, our prototype study intervention—the Agenda Gap—is comprised of an innovative, multi-media engagement intervention, developed in collaboration with youth. This intervention aims to equip youth and build capacity for them to lead meaningful policy change reflective of the mental health needs of diverse communities of youth, including those who experience structural vulnerability and who would not typically have had their voice represented in policymaking processes.

**Methods:**

This study will use a multiple case study design and mixed methods grounded in a realist approach and will be conducted in three sites across two Canadian provinces (British Columbia and Alberta). In an earlier phase of this research, we collaboratively designed the prototype intervention with youth, community and policy partners. In this phase of the study, the intervention will be implemented and further tested with new groups of youth collaborators (n = 10–15/site). Outcome data will be collected through realist qualitative interviews, validated questionnaires [i.e., Child and Youth Resilience Measure (CYRM-12), General Self-Efficacy (GSE) Scale, and the Critical Consiousness Scale (CCS)] and additional survey items developed by our study team. Analysis will focus on identification of key context-mechanism-outcome configurations to provide comprehensive insights into how this intervention works, for whom, and in what context.

**Discussion:**

This study is unique in its “upstream” focus on youth-engaged policymaking as a tool for improving the social and structural conditions that influence youth mental health across socioecological levels. Through the implementation and testing of the Agenda Gap intervention with diverse youth, this study will contribute to the evidence base on youth-engaged policymaking as a novel and innovative, mental health promotion strategy.

## Background

Mental health challenges are a leading concern for youth globally, requiring a comprehensive approach incorporating promotion, prevention and treatment within a healthy public policy framework [1]. A healthy public policy framework centers issues of health and equity in all areas of policy, and while such an approach has been identified as a policy priority across a variety of national contexts [2–7], its broad enactment has yet to be achieved. Mental health promotion is a strengths-based orientation that focuses on enhancing positive mental health for all people across the spectrum of need [8, 9] and is distinct from the prevention and treatment paradigms, which focus on addressing mental health challenge or disorder [10]. Positive mental health includes qualities such as self-esteem, the ability to effectively manage stress, and a sense of wellbeing [11, 12]. Mental health promotion is aimed at building individual and community capacity to overcome barriers and enhance mental health [13] and is described as “upstream” because it aims to alter the social and structural determinants—or “causes of causes”—of mental health (e.g., healthy child development, income and social status, social support networks, education, and culture, among others) and mental ill-health [14]. In doing so, this framework is responsive to the contextual factors that contribute to inequities that place some populations at greater risk—or conversely—are protective.

In the last few decades, the role of policy as a key mental health promotion strategy has emerged alongside a global interest in youth engagement in policy processes. Indeed, across a variety of jurisdictions, efforts aimed at facilitating youth engagement in policy have surfaced and include, for example, youth advisory committees at all levels of government. In the Canadian context, this has involved the emergence of provincial initiatives for including youth in government consultation to inform decisions about employment and child welfare, amongst others [15–17] and the development of Canada’s first national Youth Policy [18, 19]. However, while youth engagement has attracted attention across sectors and there is broad consensus that youth engagement in the design of health-related initiatives can yield positive results [20–22], there is a paucity of evidence-based strategies to support their meaningful inclusion in policy processes [10]. This is a critical oversight. Without evidence to guide youth engagement in policy, this process can be tokenistic and result in programs and services that are inadequate for youth needs. It can even contribute to worsening inequities by overlooking or intentionally excluding youth who experience structural vulnerabilities or who lack skills in this form of participation.

While there is limited scientific evidence to inform *meaningful* policy engagement, the benefits of youth engagement in policy decisions are well documented and wide-ranging. These benefits include enhanced sense of empowerment, life skills, self-esteem, and citizenship skills [23]. Additionally, youth engagement in policy processes has been shown to increase a sense of community and promote resiliency [24]. Further, the identification of issues, potentially overlooked by others, contributes to increasing decision making and solution relevancy, thus enhancing program and policy applicability, implementation, and utilization [25, 26]. Finally, when programs allow youth to address conditions in which they live, they provide participants with valuable awareness about the social and structural determinants of health that can balance individualistic, personal responsibility messaging [27] and support action across the socioecological domains that influence young peoples’ mental health [28, 29].

Recognizing the benefits of youth engagement in developing the programs and policies that affect them, this type of participation is now framed as a human right and the World Health Organization (WHO) has advised that youth “should be involved from the start as full and active partners in all stages” of programming that affects them [30]. Youth engagement in policy development—particularly those experiencing structural vulnerability and who are often excluded as a result—can deepen our understandings of youths’ experiences of mental health [31, 32] in their communities and, consequently, contribute to the development of contextually relevant and responsive mental health promotion intervention [20, 33]. Given these current priorities and identified knowledge gaps, including a paucity of resources to build capacity for youth engaged policymaking [10], our team has developed a prototype intervention—the Agenda Gap—to equip youth for meaningful policy engagement. This protocol describes our team’s anticipated contributions to the evidence base guiding youth engagement in policy and documenting our evaluation approach for this complex intervention. Given the novelty of the topic and methods, this protocol will provide valuable opportunities to elicit broader feedback and enhance the quality of this research in the current phase and beyond.

### Study aim

The study aim is to further refine and test an upstream, multi-level mental health promotion intervention—the Agenda Gap—to equip youth for meaningful policy engagement to enhance the conditions that shape the mental health of all youth, across the spectrum of need. Through the study activities, this research will make a needed contribution to the science and practice of youth-led policy engagement—a key mental health promotion strategy.

### Research objectives

1. To refine and test the prototype intervention—the Agenda Gap—to understand how the intervention works, for whom, and in what contexts.
  - To implement and test mentor training designed to build capacity to support intervention delivery, feasibility, sustainability and future scale-up through training of site-specific mentors.
  - To engage and equip youth to adapt, test and confirm the mental health promoting impacts of the Agenda Gap intervention across socioecological levels (i.e., individual, family, community, population).
2. To sustain and expand our multi-level partner network to support intervention activities and their upstream impacts as well as to inform evaluation and future scale-up.

### Intervention overview

The Agenda Gap was developed in collaboration with a diverse group of youth partners (n = 10). While this group is small, it was appropriate for facilitating meaningful engagement and co-creation of the prototype intervention. Moreover, these youth collectively brought lived expertise representing many of the identities and experiences known to contribute to mental health inequities (i.e., foster care, mental health challenges, poverty, racialization, indigeneity, and substance use leading to harms, among others). More importantly, they brought extensive expertise in advocacy, community change initiatives, and community-based research—as well as a strong commitment to actions to improve the lives of their peers.

The co-developed intervention consists of 11-weekly youth capacity-building sessions, each intended to last approximately 2 to 3 h. Youth participants will be recruited to the intervention through a variety of mechanisms, depending on the host organization or agency. For example, existing youth groups at schools or those affiliated with community organizations may participate in the intervention. Alternatively, new groups of youth may be established based on interest or shared experiences (e.g., common community, identification as an immigrant or refugee). Sessions include multi-media content and a range of applied activities and facilitated discussion grounded in principles of Social and Emotional Learning [34]. These will be co-facilitated by a youth and adult mentor at each site to support youth participants in: (a) identifying factors in their community that impact youth mental health and are amenable to change through policy; (b) developing strategies and action plans to effect relevant policy development/change; and (c) engaging with stakeholders, including policymakers, in collaborative policymaking processes to promote youth mental health and substance use outcomes.

Core intervention topics include:

- mental health promotion literacy and the social determinants of mental health
- the role of resilience in supporting youth action (case study)
- youth rights and the role for youth in identification of policy change priorities
- understanding inequities through an intersectional lens
- understanding policy and how it impacts youths’ everyday lives and mental health
- accessing and interpreting evidence to inform policy change
- campaign development/socialization of policy priorities
- policy engagement skill building—influencing systems and system actors
- tracking/monitoring policy impact (evaluation).

### Theoretical foundation for the intervention

The Agenda Gap curriculum is intended to impact youth mental health across the socioecological domains (i.e., from the individual through population levels) [34]. The socioecological model has a long history as a concept in health promotion and provides a way of considering the interplay between determinants of health across multiple levels. Our approach to developing this intervention is grounded in various theories and principles of youth engagement [35, 36], mental health promotion [29, 34], liberation psychology [23], social and emotional learning, and trauma and violence informed practice [37, 38]. Specifically, the Agenda Gap draws on Positive Youth Development, which guides meaningful youth engagement to support growth in developmental competencies, with a focus on structurally vulnerable youth. Structural vulnerability is a social sciences concept that is used to explicitly identify risk as being located in system structures, as opposed to within the individuals or their behaviours [38, 39]. Given our commitment to influencing mental health outcomes at a community or population level, we have incorporated elements of Community Youth Development, which emphasizes social justice and culminates in expanded capacity to address social inequalities. Further, our intervention is novel in its focus on mental health—as distinct from mental health challenge or disorder, which is informed by mental health promotion theory. Mental health promotion theory identifies policy as a key lever for supporting or enhancing positive mental health by shaping the conditions that influence mental health outcomes across socioecological domains [28, 35]. Additionally, theories stemming from liberation psychology, including the Theory of Sociopolitical Development [27], were incorporated as they guide us in addressing “the roots of social problems, empowerment, and the capacity to identify, analyze, and act on issues relevant to youth” [23, p. 782]. Social and Emotional Learning (SEL) was also selected as a guiding framework as it is strongly aligned with the concepts of mental health promotion and provides strategies for supporting young peoples’ competencies in the development of meaningful relationships, emotional regulation, and civic awareness, among others [40]. To promote safety of youth participants, our intervention incorporates a trauma and violence informed practice approach. Trauma and violence informed practice is a strengths-based approach that supports an interventional context that minimizes harm to participants—regardless of whether they have a known history of trauma [37]. This approach is crucial to working with populations experiencing health and social inequities [37, 38].

## Methodology

This multiple case mixed-methods study will further refine and test the prototype Agenda Gap intervention with additional groups of youth, aged 15–18, across three sites in British Columbia and Alberta, Canada. Study sites have been selected based on existing community-based partnerships and to ensure the representation of diverse youth identities including urban indigenous youth, racialized and newcomer youth, and youth who experience structural vulnerabilities. This protocol has been written using the Standard Protocol Items: Recommendations for Interventional Trials guidelines (SPIRIT).

### Study methods

**Fig. 1:**
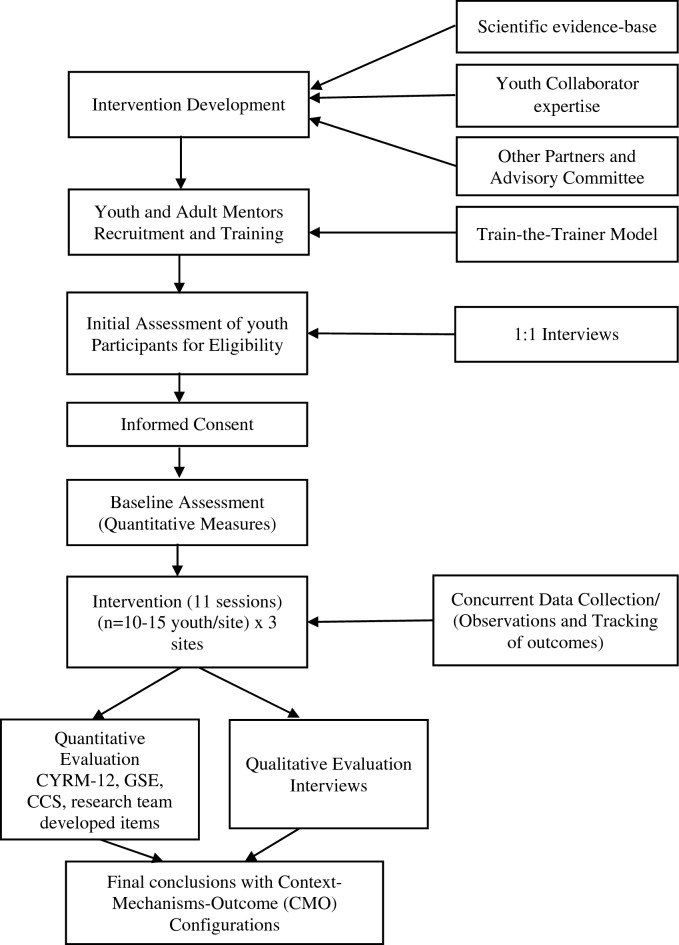
Realist evaluation: study overview

This study is grounded in a realist evaluation approach and depicted in Fig. 1. Informed by realist philosophy, this theory-driven methodology supports the identification of key context-mechanism-outcome configurations (CMOs) underpinning intervention activities to inform comprehensive insights into how the intervention works, for whom, and in what context. In realist evaluation, Context refers to the conditions (encompassing populations and social/structural conditions) that influence intervention outcomes. Generative mechanisms describe the processes (e.g., social/psychological drivers) that contribute to observed outcomes. The outcomes (intended and unintended) result from the interaction between contexts and mechanisms. This evaluation approach will improve our understanding of how the Agenda Gap intervention operates, thereby, supporting the testing and refinement of theories undergirding our intervention and enabling us to make meaningful contributions to youth mental health research agendas at multiple levels (e.g., increased evaluation/research capacity among mentors and youth collaborator participants; new insights on a methodological approach that can inform broader evaluation practice; increased understanding of functional mechanisms and relevant concepts that can be applied to other programs/contexts; identification of the necessary contextual features for effective program functioning) [41].

### Realist evaluation stages

**Fig. 2:**
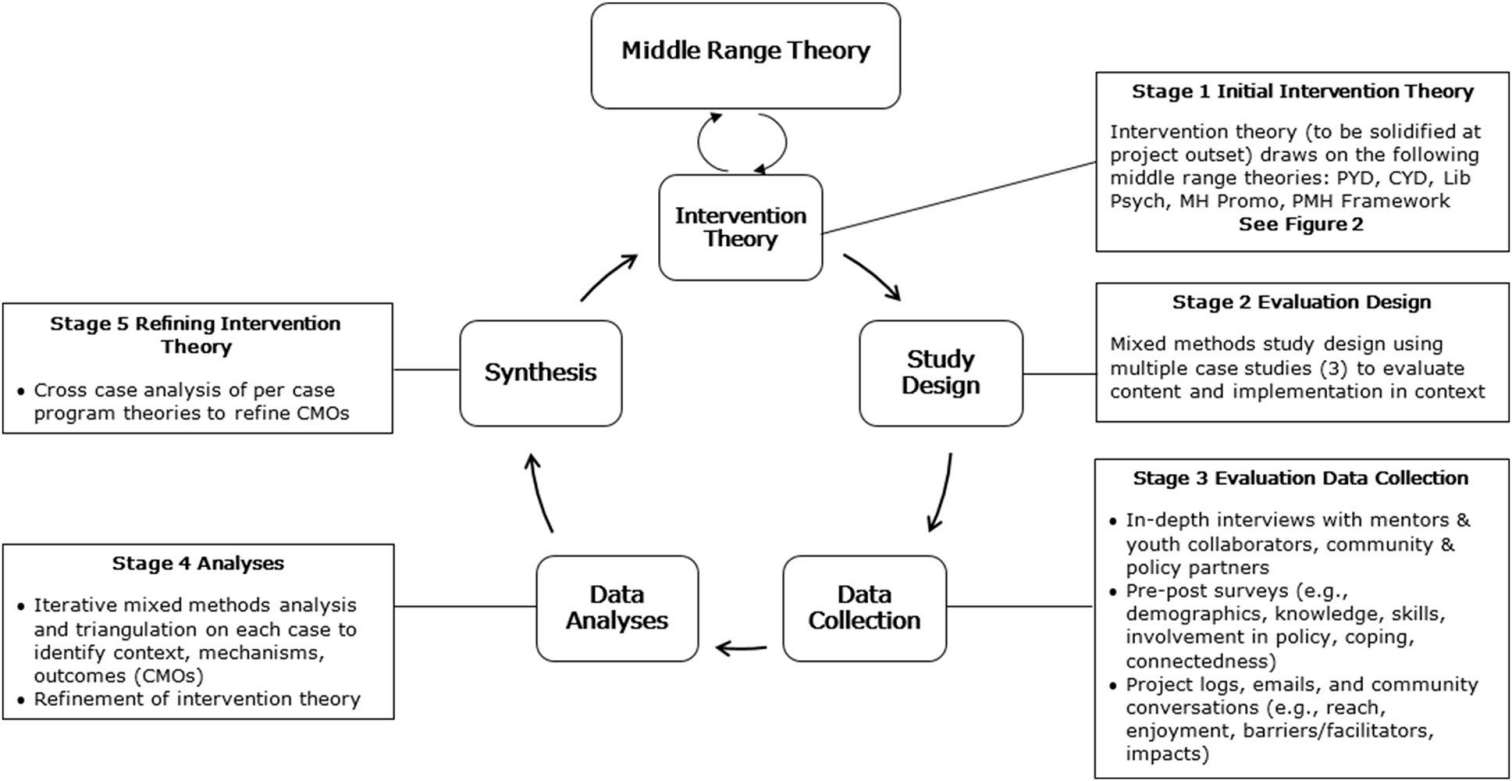
Evaluation cycle (Adapted from [42])

Realist evaluation is comprised of five stages [42], which are detailed for our study below (Fig. 2).

#### Stage 1—Initial intervention theory

**Fig. 3:**
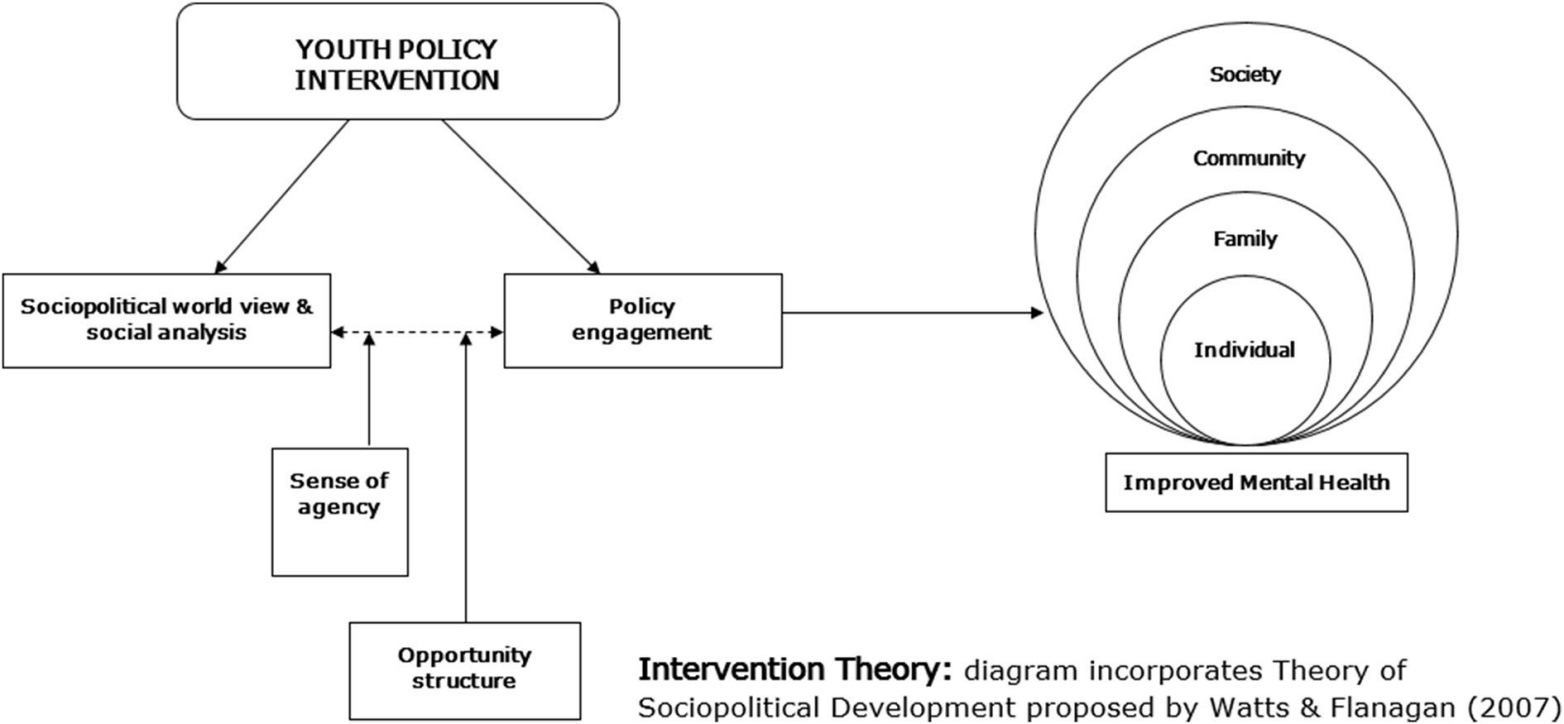
Initial intervention theory

As per realist evaluation methodology, our research process began with a review—in our case, this took the form of an environmental scan to identify existing resources and evidence informing practices to equip youth for meaningful policy engagement [10]. This review, along with theories of youth development, mental health promotion, and liberation psychology identified above, have informed our “Initial Intervention Theory” (IIT) (Fig. 3). Guided by the realist evaluation approach, this IIT will be tested and refined though our study activities. Specifically, the IIT as well as the various elements of the intervention itself will be described through a series of “program theories”, or “if/then” statements that are intended to explain the causal process believed to underpin the intervention activity effects. For example, we hypothesize that financial compensation for our youth collaborators is a key element that will contribute to intervention effects. As such, we have developed and will test this as a program theory framed as, “if youth who have historically felt that their voices are not heard [Context] are financially compensated for their collaboration in the Agenda Gap intervention [Mechanism], then they will feel valued, committed and motivated. This will lead to reduced barriers to participation, sense of co-ownership and sustained involvement [Outcome].”

Our IIT builds on the Theory of Socio-political Development [27], which describes youth policy engagement as a product of social awareness and action, moderated by perceived agency and existing opportunity. We have advanced this theory and suggest that our Agenda Gap intervention will build critical consciousness—or the ability to recognize and act on issues of inequities. In turn, we hypothesize that this action will generate an enhanced sense of agency amongst participants, contributing to further opportunities for meaningful engagement within policy contexts—with mental health promoting impacts across the socioecological domains. For example, this intervention holds the potential to influence study participants directly, by equipping them with new knowledge and skills pertaining to mental health and policy. Further, through the engagement process, we anticipate impacts on youth, policymakers and others living in communities where policy change efforts are initiated.

#### Stage 2—Study design and recruitment

We are using a multiple case study design across three sites, with one case consisting of each site’s team (e.g., mentors, youth collaborators, site organization) and context. Each case will enable cross-case comparisons to identify common and distinct CMOs and their configurations [43–45]. This will allow us to test the intervention and program theories across diverse contexts and youth populations, with respect to mechanisms theorized to produce expected outcomes.

#### Mentor recruitment and training

This study will begin with the identification and training of youth and adult mentors across three sites. Mentors will be identified through our partner networks (i.e., community organizations, non-profits, health authorities, School Boards) and bring experience in working with youth and of engaging in policy change initiatives. In future iterations, additional partnerships will be developed to support intervention scale-up. Our train-the-trainer model will involve approximately 12 h of training delivered by a Community Engagement Specialist. This training will equip mentors to facilitate engagement with youth collaborators, while also building leadership skills and growing local capacity for intervention sustainability. Mentor training will include how to:

- promote social and cultural safety among youth from diverse contexts and backgrounds
- model or bring lived expertise of policy engagement or advocacy
- enable shared decision making with youth for meaningful youth engagement
- incorporate principles of trauma and violence informed practice
- be inclusive of diverse learning needs.

To ensure mentors have the needed level of substantive knowledge to support implementation of the intervention, a Facilitator’s Guide has been developed and contains step-by-step details of the curriculum content and strategies. This approach was selected over a manualized approach to intervention delivery to promote flexibility, while still providing consistent guidance across sites. As mentors will be an important data source, all mentors will provide written informed consent prior to participating in study activities.

#### Youth collaborator recruitment

A particularly novel element of our approach is that we are committed to engaging a diversity of youth, aged 15–18 years, from structurally vulnerable communities. Policy participation for such youth has remained largely inaccessible due to intersections of health and social inequities that operate to exclude certain groups from contributing (i.e., youth who have experience with mental health services, live in poverty, are in the care of the child welfare system, or who are Indigenous, newcomer/immigrants, or LGBTQ2+). Our equity-oriented approach and strong community and policy partnerships will enable us to engage our youth participants to better reflect the lived experiences of youth who have some of the greatest mental health needs [46, 47]. By working with youth who have historically not had opportunities for engagement and leveraging our partner networks to influence policy, we can contribute to more equitable contexts, which are supportive of improved mental health outcomes for all. Recruitment will be facilitated by our community partners, with interested youth encouraged to contact the research team. All applicants will be interviewed to assess eligibility and interest in becoming a youth collaborator on the study as well as to ensure the inclusion of diverse youth identities (e.g., age, gender, social location). A total of 10–15 youth will be selected (after obtaining informed consent) as collaborators for each of the three sites. This position will be a paid position to appropriately acknowledge the expertise and contributions that these youth will make to the intervention and research process.

Our project also includes ongoing partnerships with adults who interact with and provide services to youth (e.g., youth workers, policymakers, educators, and health and social service providers). This engagement builds relationships through which knowledge and perspectives are shared; and understandings, beliefs, and behaviors shifted to contribute to more equitable and mentally healthy communities [20]. For example, one of the critiques in the youth engagement literature is that youth tend to be the sole intervention target. Instead, efforts need to include strategies to support relevant adults in appreciating and recognizing the various ways that youth are already “engaging” (which may differ from adults’ preconceived notions) and shift systems to be more responsive to diverse forms of engagement [48].

Youth and their communities, adult and youth mentors, and policymakers, will be involved in all phases of intervention refinement, implementation and testing through direct engagement in project activities and membership on Advisory Committees that guide project processes, partner expansion, recruitment, curriculum refinements, youth mentorship, youth meetings with policymakers, and other youth identified activities.

#### Stage 3—Data collection methods

A mixed methods approach to data collection will allow for comprehensive measurement/tracking of inputs (i.e., suggested intervention refinements), implementation process and intervention outcomes. Quantitative and qualitative data will support the characterization of CMOs and community perceptions of intervention impact.

Quantitative surveys will be used to gather data pre- and post-intervention to examine the impact of the intervention at multiple levels. Specifically, these surveys will include demographic questions to explore how different factors may be related to outcome patterns, an assessment of knowledge gains, standardized tools measuring key hypothesized outcomes (i.e., resilience, self-efficacy, and critical consciousness) and will collect indicators or metrics of policy engagement and change.

Standardized quantitative tools include:

- Child and Youth Resilience Measure (CYRM-12): The CYRM-12 is a brief self-report scale that measures youth resilience. It has been used with youth in a variety of contexts and has good content validity and reliability (Cronbach’s alpha = 0.840) [49].
- General Self-Efficacy (GSE): This 10-item scale has been used widely among youth and adult populations to assess self-beliefs for dealing with various life challenges. GSE has been demonstrated to measure a unitary concept with the good psychometric properties [50, 51].
- Critical Consciousness Scale (CCS): The CCS is a validated, 22-item scale that measures the capacity of structurally vulnerable youth to reflect on and analyze their social conditions and engage in action for change [52].

In addition to standardized tools, we will develop items to quantitatively assess mental health promotion literacy and indicators of positive mental health.

Given the complexity of policy influence and the challenge of linking intervention activities with policy change, in addition to the ideal “hoped-for” policy impacts, we will collect data across a continuum of policy influence processes (e.g., precursors to policy change) [53]. Additionally, data on the economic inputs or costs associated with intervention delivery will be tracked for future feasibility and sustainability considerations.

Qualitative realist interview methods will be used post-intervention to collect varying perspectives from youth, mentors, community partners and other stakeholders about the intervention, which will complement structured surveys in verifying and refining the program theories. Qualitative realist interviews are a key source of theory refinement and discovery of underlying mechanisms within realist evaluation. They are also a valuable tool for surfacing youth and community voices and for assessing, from participant perspectives, the nuanced ways in which the intervention contributes to policy change and risk and protective factors, at the individual through population levels [54, 55]. Project logs will also be maintained by the research team to capture observations of community resources and dynamics, including those unspoken during intervention sessions, and to inform intervention refinements.

#### Stage 4—Data analyses

Quantitative survey data will be entered into SPSS to facilitate statistical analyses. Descriptive statistics will be calculated for each question to determine the effectiveness of the intervention for addressing each of the constructs represented in the survey. Paired t-tests will be used to assess pre- and post-intervention impacts (e.g., does resilience increase post-intervention?). When sample sizes permit, ANOVA will be employed to examine mean scores and change across sites to gain an understanding of impact in varying contexts.

All qualitative data (i.e., individual interviews and project log data) will be transcribed and uploaded to NVivo 12 to facilitate thematic analyses that will generate high-level themes, representing findings within and across transcripts [55]. During later intervention engagement sessions, youth collaborators will participate in collaborative analysis, an iterative process with the research team, who will produce summaries of identified themes and engage youth collaborators to reflect and extend interpretations. Triangulation of data sources (quantitative, qualitative and cross-site) will be undertaken to: (a) gain a comprehensive understanding of the impact of the intervention; (b) illuminate CMOs; (c) examine regularities or areas of contradiction between data sources; and (d) as an approach to analytic and procedural rigor to support the validity of findings [56]. Analyses will be concurrent and ongoing throughout the study process to support rich and detailed analyses that are responsive to emerging understandings [57].

Additionally, an intersectionality lens will inform analyses to assess outcomes by contextual and identity factors. This is consistent with realist evaluation, which seeks to represent the multiple and intersecting ways that sex, gender, socioeconomic status, health status, ethnicity, and other factors shape participants’ experiences with mental health and responses to the Agenda Gap intervention. Data monitoring and management will be conducted by the core research team. At least two researchers or research manager will ensure the data quality.

#### Stage 5—Synthesis

Final in-case analyses and comparisons with original IIT and program theory predictions will help to determine CMO configurations with the greatest potential [58]. During this stage, final conclusions will be drawn about *what works, for whom, and in what context* (e.g., hypotheses about how various contexts facilitated or inhibited generative mechanisms). Results will refine the program theories, which, in turn, will guide intervention refinement and contribute to the broader mental health promotion research and practice agenda.

#### Sustainability planning

The research team will work with youth collaborators and policy partners to negotiate an appropriate mechanism to support sustained youth policy engagement (e.g., Community of Practice; youth champions; dissemination plans for Agenda Gap intervention). A formal sustainability plan will be developed in future project phases. Policy partners will also be enlisted to help to disseminate project findings and outputs to build awareness to support meaningful youth engagement in the policy setting.

## Discussion

Extending our program of research, this study will address Canada’s youth mental health shortfalls by contributing new evidence on upstream processes and intervention for engaging youth in developing policies to promote mental health. Importantly, this intervention diverges from other initiatives already in place and responds to calls from researchers and health advocates to implement a critically-driven population health approach to addressing mental health—one that incorporates promotion within a healthy public policy framework [1]. While this has been identified as a policy priority in Canada [1–3, 59] and beyond, it has yet to be widely implemented. Further, while there is an extensive literature on mental health interventions—both prevention and treatment—evidence on mental health promotion among youth is more limited and has not yet focused at a policy level [60, 61].

Crucially, international recommendations are aligned with policy intervention as mental health promotion through common aims: to promote and support healthy lives for all children and youth, take subjective wellbeing seriously, and prioritize equity in child health promotion agendas [62]. As mentioned above, through forging collaborative partnerships with groups not typically included in policy and practice consultations, our project will be unique in focusing on so-called ‘disengaged’ youth and on community contexts in which policy participation remains largely inaccessible due to structural vulnerability. Further, this phase of our research on youth policy engagement provides opportunities to expand and strengthen our community and policy partnerships. By engaging existing partners as well as new youth collaborators, this study holds potential to demonstrate real-world impact, led by youth. In turn, it is positioned to draw interest from external groups to facilitate future partnerships and support scale-up of the Agenda Gap intervention across Canada and beyond.

Despite the new evidence that this study will produce, there are potential limitations that warrant discussion. Specifically, given the preliminary nature of this phase of work, sample sizes for quantitative analyses will be small, though of sufficient power to detect a medium effect size (p < 0.05, two-tailed). Power will be less of an issue in future phases as additional sites are added, including the potential to bring the intervention to school contexts where large numbers of youth could benefit from the intervention. Additionally, as a quasi-experimental study, we will not be able to make claims about the causal relationships between study variables. However, realist evaluation is well-suited for this phase of work and for gaining a nuanced understanding of how the intervention works within context. Finally, in our experience of conducting community-based youth mental health research we have observed a relationship between affluence and engagement, where youth from more vulnerable families and marginalized neighborhoods can be more challenging to engage. Our partnerships with community organizations within each site will help to mitigate this by facilitating trusting relationships with potential youth collaborator participants. Anticipating these challenges and identifying strategies to mitigate these will position us well for ensuring the success of this project.

## Conclusions

Mental health is a priority issue for youth globally, requiring a comprehensive approach encompassing promotion, prevention and treatment. While mental health promotion offers a promising, strengths-based orientation, it remains understudied. Utilizing participatory approaches and realist evaluation, this study holds the potential to make a meaningful contribution to the science and practice of mental health promotion to enhance youth mental health outcomes across the socioecological domains, through youth engagement in policy making processes.

## Data Availability

Not applicable. Data sharing is not applicable to this article as no datasets have been generated or analyzed for the described study.

## Abbreviations

CCS: Critical Consciousness Scale
CMO: Refers to context, mechanisms and outcomes in realist research
CYRM-12: Child and Youth Resilience Measure
GSE: General Self-Efficacy
IIT: Initial Intervention Theory
LGBTQ2+: Lesbian, gay, bisexual, transgender, queer/questioning
